# Design and implementation of a portable colposcope Mueller matrix polarimeter

**DOI:** 10.1117/1.JBO.25.11.116006

**Published:** 2020-11-15

**Authors:** Mariacarla Gonzalez, Karla Alejandra Montejo, Karl Krupp, Vijaya Srinivas, Edward DeHoog, Purnima Madhivanan, Jessica C. Ramella-Roman

**Affiliations:** a Florida International University, Biomedical Engineering Department, Miami, Florida, United States; b Public Health Research Institute of India, Mysore, Karnataka, India; c University of Arizona, Mel and Enid Zuckerman College of Public Health, Department of Health Promotion Sciences, Tucson, Arizona, United States; d Optical Engineering and Analysis, Long Beach, California, United States; e University of Arizona, College of Medicine, Department of Medicine, Tucson, Arizona, United States; f University of Arizona, College of Medicine, Department of Family and Community Medicine, Tucson, Arizona, United States; g Florida International University, Herbert Wertheim College of Medicine Cellular Biology and Pharmacology, Department of Ophthalmology, Miami, Florida, United States

**Keywords:** collagen, Mueller matrix, colposcopy, polarimetry

## Abstract

**Significance:** Mueller matrix polarimetry can provide useful information about the function and structure of the extracellular matrix. A portable and low-cost system could facilitate the clinical assessment of cervical anomalies in low-resource settings.

**Aim:** We introduce a low-cost snapshot Mueller matrix polarimeter that does not require external power, has no moving parts, and can acquire a full Mueller matrix in ∼1  s, to conduct a feasibility study for cervical imaging in the low-resource setting.

**Approach:** A snapshot system based on two sets of Savart plates, a ring illuminator with polarizing elements (generating four polarization states), and one camera is introduced. Stokes vectors are formulated to recover the polarization properties of the sample. Then, using Mueller matrix decomposition, the depolarization and retardance information is extracted.

**Results:** We report the results on 16 healthy individuals (out of 22 patients imaged), whose Pap smear showed no malignant findings from mobile clinics in rural region of Mysore, India. The depolarization and retardance information was in agreement with previous reports.

**Conclusions:** We introduce an imaging system and conducted a feasibility study on healthy individuals. This work could futurely translate into diagnostic applications to provide a quantitative platform in the clinical environment (e.g., cervical cancer screening).

## 1 Introduction

Cervical cancer is the fourth most common cancer in women worldwide, with an estimated half a million new cases and 311,000 deaths each year.^1^ Developing countries suffer about 84% of the global burden of disease and 80% of the mortality due to a lack of effective screening programs. The hardest-hit regions are among the world’s poorest with incidence rates over 35 per 100,000 women compared with 3 per 100,000 women or lower in North America and Europe.^2^ Because the disease progresses over many years, an estimated 1.4 million women worldwide are living with cervical cancer, and two to five times more—up to 7 million—may have precancerous conditions that should be identified and treated.^3^ While several prophylactic human papillomavirus (HPV) vaccines are now available in more than 100 countries for primary prevention,^4^ they do not target all 15 high-risk HPV types, ergo there is still a need for screening.^5^ Moreover, due to cost-effectiveness issues of vaccination in low- and middle-income countries, it is often seen that the only available prevention method is regular screening and treatment of precancerous lesions.^6^

In India, cervical cancer is the second most common cancer among women aged 15 to 44 years.^7^^,^^8^ There are about 122,844 new cases and 67,477 deaths annually among the approximately 432.2 million women at risk.^8^^,^^9^ As with other low-income countries, traditional cytology-based diagnostics are largely impractical for population-based screening because of cost, inadequate infrastructure, lack of skilled health care workers, and laboratories.^10^ While guidelines for population-based screening have been established for more than a decade, it has been estimated that less than 4% of at-risk women have currently been screened for cervical cancer.^7^ Several states in India have initiated pilot programs examining the effectiveness of visual inspection with acetic acid (VIA) for diagnosis of cervical neoplasia.^11^^,^^12^ In India, VIA would be considered the current best alternative, although it has several important limitations with 71.8% sensitivity, 79.4% specificity, positive predictive value of 16.7%, and negative predictive value of 99%.^13^ The high potential for false positives in VIA is a great concern^14^^–^^16^ since it can lead to excessive testing and overtreatment including unnecessary colposcopies, biopsies, cryosurgery, and hysterectomies as evinced by programs in Nepal and India.^17^^,^^18^

Low-cost optical technologies, such as the high-resolution microendoscope (HRME),^19^^–^^21^ the point of care tampon-based digital colposcope (POCKeT colposcope),^22^ or the cellphone-based MobileODT system,^23^ are being proposed but have limitations. HRME is a point measurement (∼500  μm in sampling size) that is still guided by physician expertise, whereas POCKeT and MobileODT focus only on acquiring digital image and still require expert review of the data^22^^,^^23^ although machine learning approaches are being tested at present.

The cervix is composed of structural tissue exhibiting birefringence^24^^,^^25^ arising by its molecular structure, as well as its very ordered arrangement within the stroma. It consists of ∼70% collagen fibers, elastic fibers,^26^^,^^27^ and a ground mixture of biomolecules (e.g., proteins and nucleic acids). The circumferentially aligned cervical collagen structure^28^^–^^31^ found around the os can be monitored with polarimetric techniques, such as Mueller matrix polarimetry, and deviation from the standard ordered structure can be used to pinpoint pathological areas.^32^ A Mueller matrix completely characterizes the polarimetric properties of a sample.^33^^,^^34^ Using Mueller matrix decomposition (MMD) (as proposed by Lu and Chipman^35^), we can obtain three canonical matrices $M=M_{\Delta }M_{R}M_{D}$: a diattenuator matrix $M_{D}$, $M_{\Delta }$ accounting for the depolarizing effects of the material, and a retarder matrix $M_{R}$. Furthermore, the resulting matrices can be analyzed to yield quantitative medium properties^36^ that have demonstrated useful diagnostic power.^37^ Of particular relevance to this study is the angle α, which is directly related to the orientation of the long axis of the collagen bundles in the tissue, and the optical retardation R, which is related to collagen density.

Mueller matrix imaging (MMI)^38^^–^^41^ has been proposed as an alternative to standard screening by several groups and focuses on subtle changes in cervical collagen structure typical of cervical precancerous lesions to provide a quantifiable map of cervical alteration in the ectocervix. For example, differences in retardance and depolarization have been shown in normal versus pathologic states.^32^^,^^37^^,^^38^ A recent *ex-vivo* study utilizing MMI has showed a sensitivity and specificity of 83% of normal versus high-grade lesion tissues,^39^ higher than the previously mentioned VIA testing. Our group has used MMI to target the fibrous ultrastructure (i.e., collagen) organization of the cervix.^42^ We have developed a clinical Mueller matrix system^43^^,^^44^ based on a standard colposcope with high sensitivity to the cervix ultrastructure. We have tested the system on healthy volunteers, as well as have measured collagen arrangement and distribution non-invasively, and have positively compared our findings to other microscopic techniques, such as optical coherence tomography.^42^

This paper describes the extension of the aforementioned study with the realization of a portable snapshot system based on Savart plates that can be deployed in low resource settings. We hypothesize that the use of this MMI system can provide fast-acquired quantitative images of the cervix that can be used during the cervical screening process to provide feedback by identifying probable pathologic areas. A pilot study introducing the potential clinical use of the device is presented. This work can translate to improving cervical cancer screening by providing a quantitative platform that is low cost and portable, which could in the future increase the diagnostic power of VIA and other screening modalities.

## 2 Methods

**Fig. 1:**
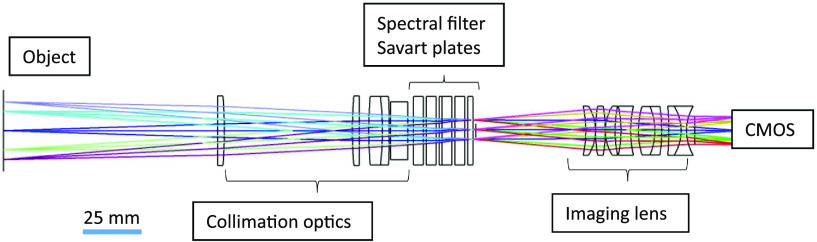
Optical layout of the PSA portion of the system.

The snapshot Mueller Matrix polarimeter consists of two different elements: a polarization state generator (PSG) and a polarization state analyzer (PSA). The PSA is designed to have a field of view of 30 mm, operating wavelength of 633 nm, and a magnification of 0.5. In the PSA, Savart plates divide the light into four separate paths, each with intensities proportional to the polarization information of the object (Fig. 1). The four separate channels are recombined by an imaging lens onto the camera creating a spatial interference pattern.

**Fig. 2:**
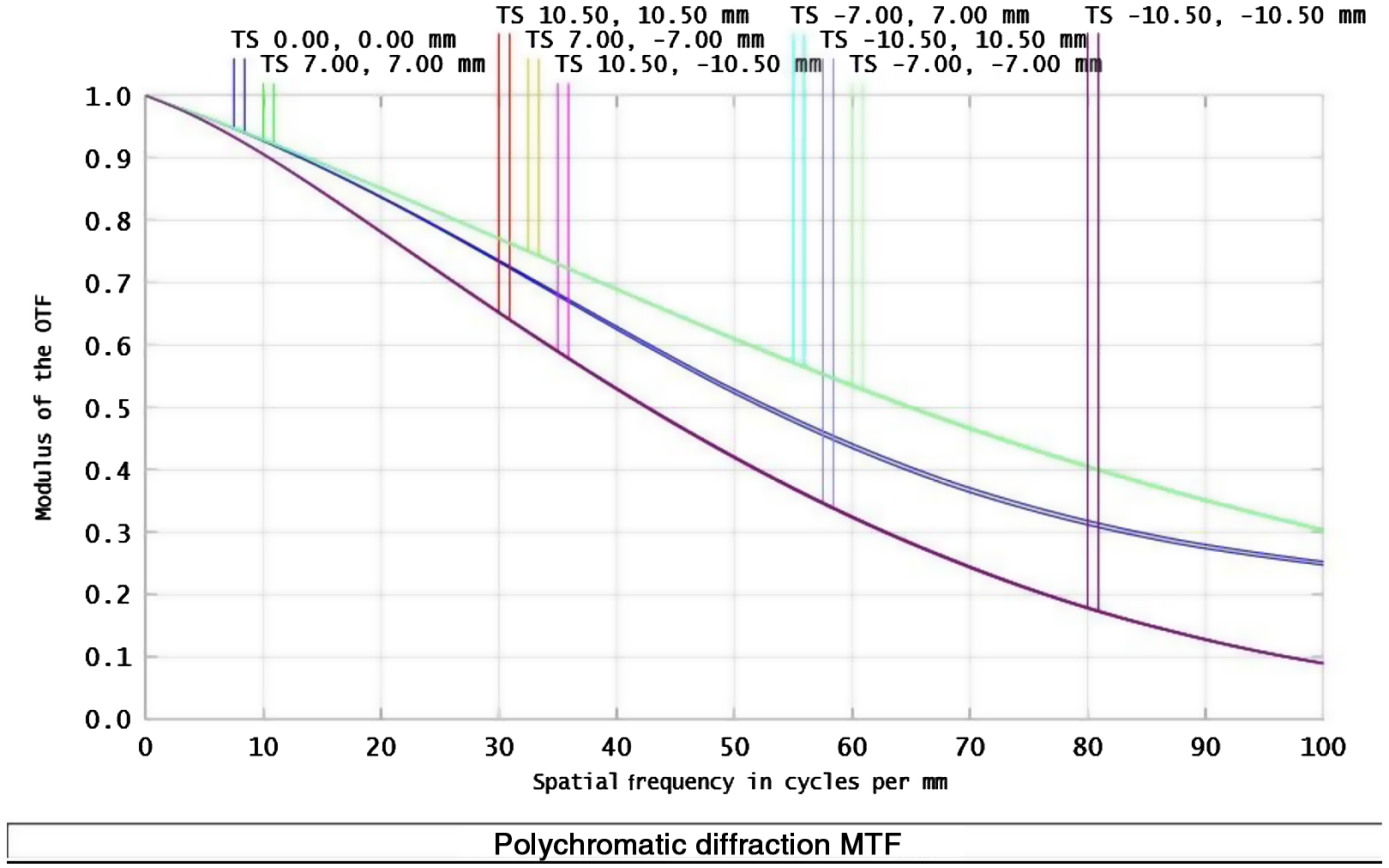
MTF of the Savart polarimeter.

The contrast of these fringe patterns is a function of the modulated transfer function of the optics and the polarization properties of the object (Fig. 2). To recover the polarization properties of the object, different reconstruction methods can be utilized.^45^^–^^47^

In one method, a Fourier transform is performed on the image. Applying spatial filters and an inverse Fourier transform results in an image of the polarization information of the object. The process is described in detail elsewhere.^45^^,^^46^ A second method, the sliding reconstruction approach,^47^ is also used. Both methods require the acquisition of calibrations images: 0 deg and 45 deg linearly polarized beam for the Fourier method and 0 deg linearly polarized, 45 deg linearly polarized, and right circularly polarized for the sliding reconstruction method.

Our system consists of four calcite Savart plates 25  mm×25  mm×3.72  mm (United Crystals LLC) with 20/10 surface quality; parallelism less than 3 arc min and AR coating (angle of incidence 0 deg to 30 deg; $R_{\text{avg}}<0.5\%$ for wavelengths 500 to 800 nm). A ½ achromatic wave plate (Thorlabs Inc.), a 50 mm EFL imaging lens (MLV 50M1, Thorlabs Inc.), and a high-sensitivity USB 3.0 complementary metal oxide semiconductor (CMOS) Cameras with Global Shutter (DCC3240C, Thorlabs Inc.) capable of 60 frames per second at full resolution 1280  pixels×1024  pixels.

**Fig. 3:**
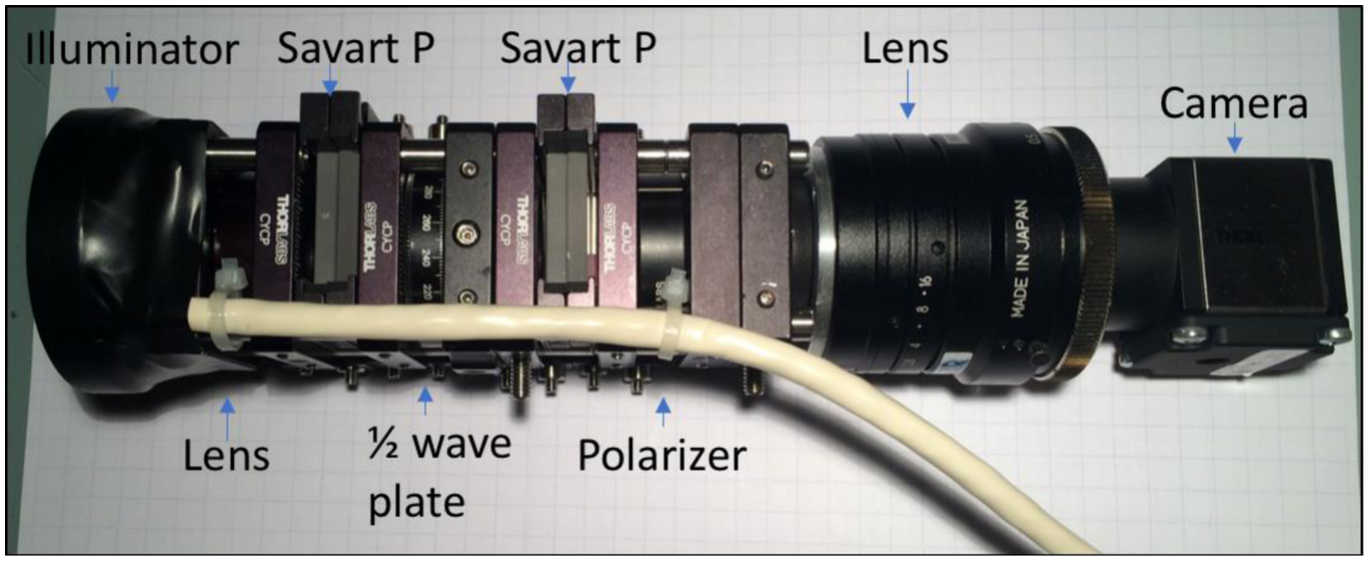
The snapshot Mueller matrix polarimeter.

An image of the full system is shown in Fig. 3. Theoretically, this system can resolve spatial frequencies between 70 and 100  lp/mm on the detector (Fig. 2). These frequencies correspond to features ranging from 20 to 30  μm on the object, which is well within the range needed to resolve the features of interest.

The snapshot system can acquire one full Stokes vector within one snapshot. Since we are interested in obtaining a full Mueller matrix, four different states of input polarizations are necessary. In previous work, three linear states (0 deg, 45 deg, and 90 deg to the reference plane and right circularly polarized) have been utilized and shown to be optimal.^48^

**Fig. 4:**
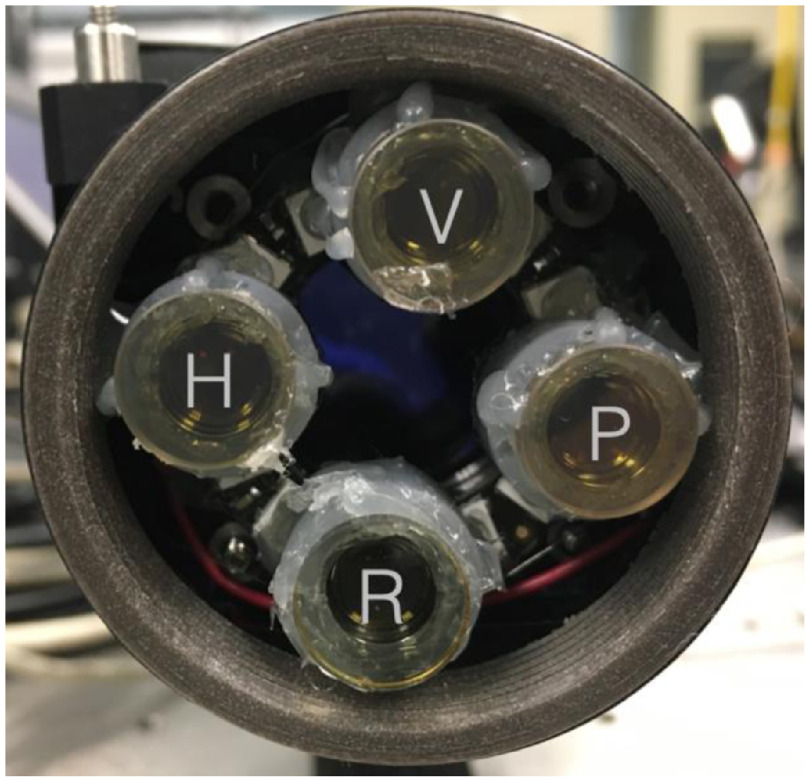
Polarization state generator. H is a horizontally aligned polarizer (with respect to the reference frame, optical bench), V is a vertically aligned polarizer, P is a 45 deg aligned polarizer, and R is a circular polarizer.

The requirement of our system to be portable, computer controllable, and easy to use by non-experts has led to the choice of a preassembled set of light-emitting diode (LED) for our PSG (Fig. 4).

A NeoPixel Ring (Adafruit, New York) with 16 red, green, and blue LED with integrated drivers was used in this system. Four LEDs were chosen on the ring and were paired with a small diffuser and cellphone lenses. Overlapping spot size of 3 cm was then achieved at a distance of about 10 cm for each LED. The LEDs spectral bandwidth at the operating wavelength of 633 nm was 10 nm full width half max (FWHM). The LEDs were connected to an Arduino Mini and a custom driver was developed to control the board through MATLAB (MathWorks, Natick, Massachusetts). Given the small dimension of the Mini, it could be integrated into a cable.

A graphical user interface was designed in MATLAB to control acquisition and illumination. The program operates in two different modes. In focusing mode, all four LEDs are activated at half of their power setting and the camera acquires at 60 frames per second. Once appropriate focus onto the cervix is achieved, the acquisition mode begins by switching off all LEDs. Then, each LED is activated in sequence and after each activation, an image is acquired. Finally, the four images are combined into a stack and saved without any filtering or manipulation. Total acquisition time is about 1 s.

Data acquired with the system are analyzed in post-processing. Mueller matrix images are decomposed with a process illustrated by Lu and Chipman.^35^ Retardation, depolarization, attenuation, and orientation images are created.

### 2.1 Image Processing

Data analysis was performed primarily using the Fourier reconstruction method. A second methodology known as sliding reconstruction method was also explored. Both methods are illustrated below.

#### 2.1.1 Fourier reconstruction

In this reconstruction method, the fringe coded image is Fourier transformed^49^ and the extracted amplitudes and phases are used to determine the Stokes parameters.

**Fig. 5:**
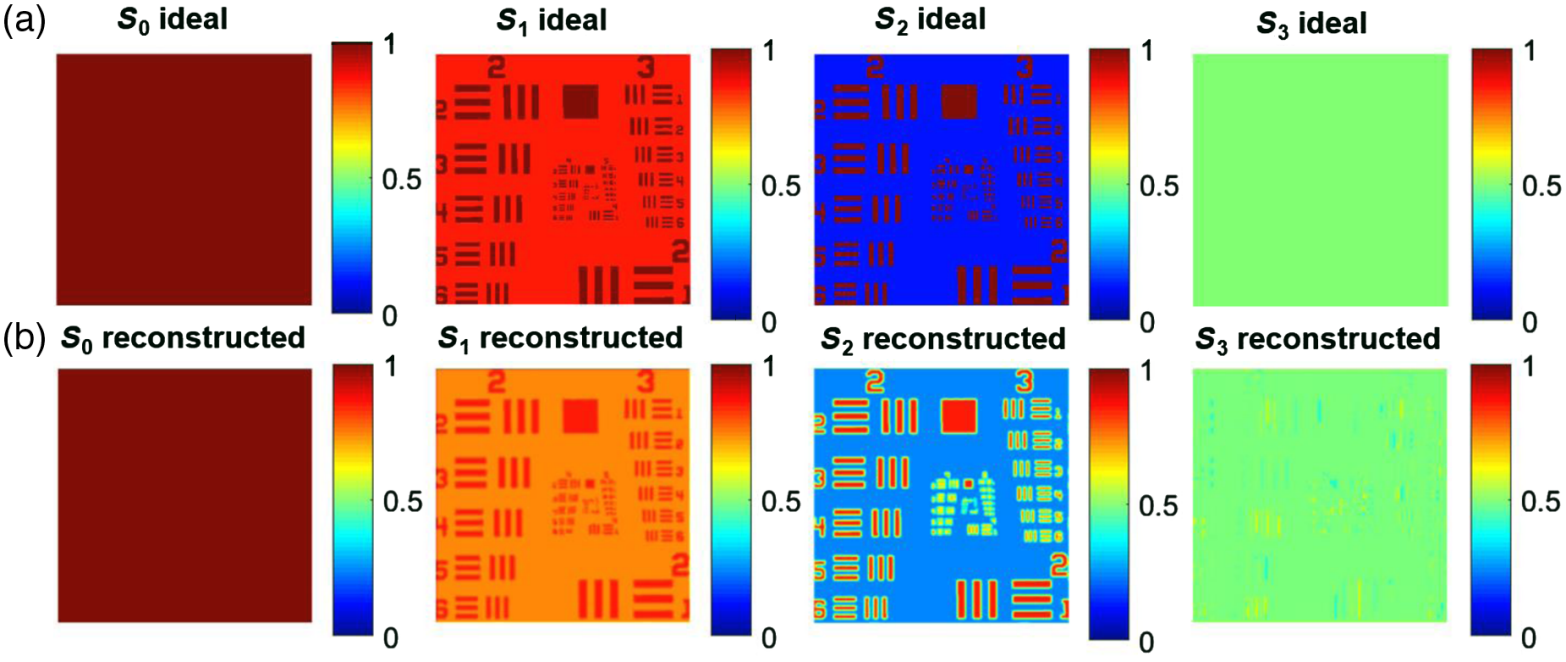
(a) An input Stokes vector was created with a USAF target. The status of polarization corresponds to a linear orientation of 22.5 deg for the background and 0 deg for the lines. (b) The Stokes vector after reconstruction. There are still visible artifacts (traces from the target) where the Fourier reconstruction failed.

Solving for the FFT of the output signal [Eq. (1)], a set of spatially independent values each carrying polarization information is observed. Many examples of this approach with ideal input Stokes vectors can be found in the literature.^45^^,^^46^^,^^50^^,^^51^ Here, we focus on a United States Air Force 1951 (USAF) target. Some of the artifacts associated with the methodology, such as errors in reconstruction in the presence of edges, can be noted in Fig. 5, e.g., $S_{3}$ reconstructed shows traces of the target meanwhile $S_{3}$ ideal does not.

The intensity I of the interference pattern relates to the incident Stokes vectors according to the following equation:^52^

$$I(x,y)=\frac{1}{2}S_{0}+\frac{1}{2}S_{1}\;\cos[2\Omega (x+y)]+\frac{1}{4}|S_{23}|\cos[2\pi (2\Omega )x-\arg(S_{23})]-\frac{1}{4}|S_{23}|i\;\cos[2\pi (2\Omega )y+\arg(S_{23})]S_{23}=S_{2}+iS_{3}\text{ and }\Omega =\frac{\Delta }{\lambda f},$$ (1)

Ω is the frequency of the spatial modulation, λ is the wavelength, Δ is the sheer distance of the Savart plates, and f is the focal length of the lens closer to the camera.

**Fig. 6:**
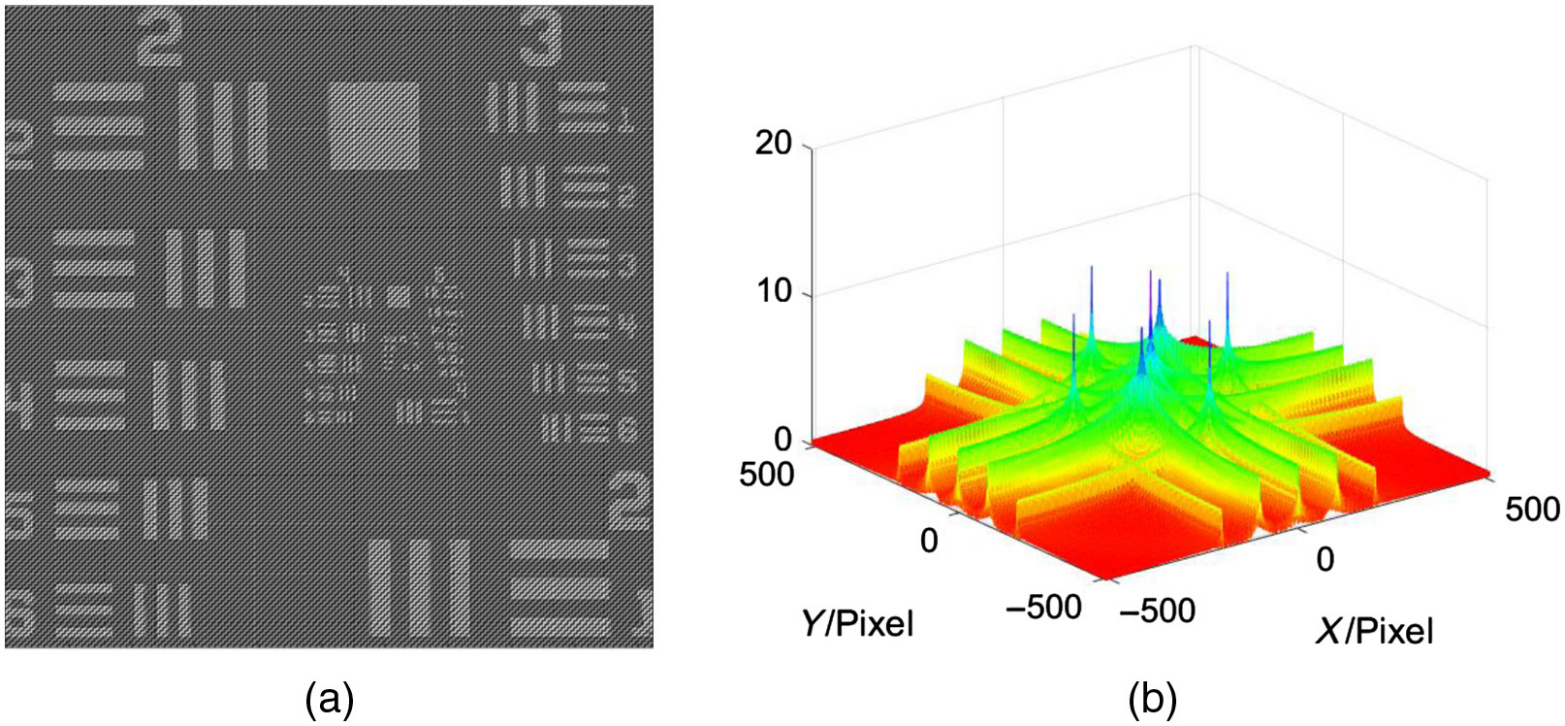
(a) Interferogram associated with the USAF test target as seen by a camera sensor, (b) Fourier transform of the interferogram of the USAF target, featuring the peaks (along with the full frequency profile) where the Stokes vectors are spatially encoded within the interferogram.

This image I is shown in Fig. 6(a) together with its two-dimensional Fourier transform [Fig. 6(b)].

**Fig. 7:**
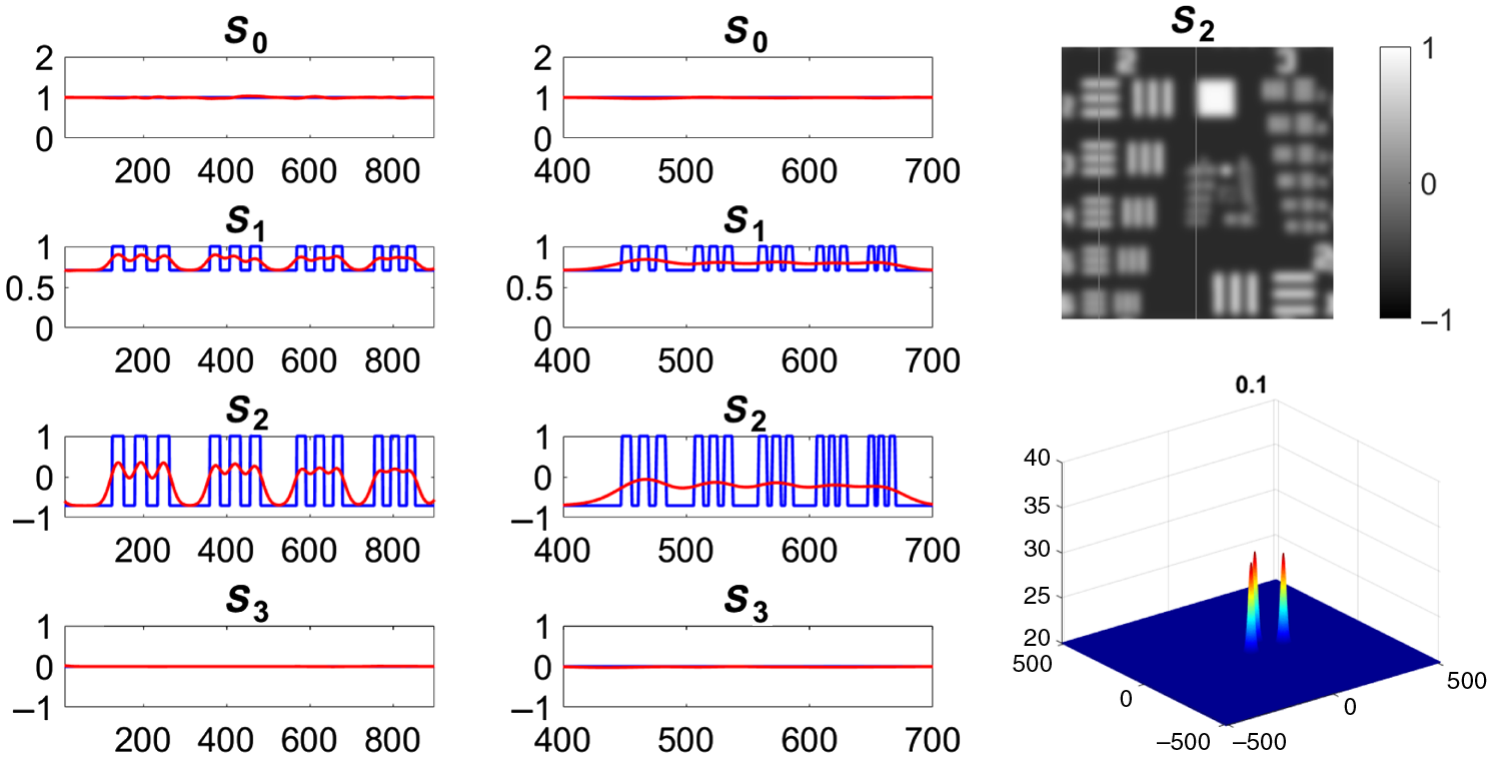
Filter design changes both the quality of the reconstructed Stokes vector and image resolution. Two traces (red and blue) are shown on the figure displaying differences in the original Stokes vector and the reconstruction (lines are shown in S2 image). Two examples are shown, one for the largest (first column) and smallest (second column) block of resolution dashes in the USAF target using the same filter. The filter appearance is shown in the bottom right of the figure as the bandwidth changes (Video 1, MP4, 2.55 MB [URL: https://doi.org/10.1117/1.JBO.25.11.116006.1]).

The spatial position of the four Stokes element vector is known and depends on the source wavelength, the thickness of the Savart plates, and the focal length of the imaging lens. A filter can be designed to extract the Stokes parameters in the Fourier domain for each state of polarization. This application utilizes three Gaussian filters to extract Stokes vectors $S_{0}$, $S_{1}$, and $S_{23}$, which is applied to the interferogram in Fig. 6(b). The Gaussian filters and effect of the filter bandwidth in relation to the Stokes vector output can be seen in Fig. 7. The filtered image is then inverse Fourier transformed and normalized.

The normalization utilizes two reference images: 0 ($S_{\mathrm{ref}0}$) and 45 deg ($S_{\mathrm{ref}45}$) linear polarizations. The reference images must undergo the same reconstruction process as previously mentioned. The final Stokes vectors, after reconstruction and normalization, will be as shown in Eqs. 2(a)–2(d).

$$S_{0}=ℜ(\frac{S_{0}^{*}}{S_{\mathrm{ref}0}}),$$ (2a)

$$S_{1}=ℜ(\frac{S_{1}^{*}}{S_{\mathrm{ref}0}}),$$ (2b)

$$S_{2}=ℜ(\frac{S_{23}^{*}}{S_{\mathrm{ref}45}}),$$ (2c)

$$S_{3}=ℑ(\frac{S_{23}^{*}}{S_{\mathrm{ref}45}}).$$ (2d)

The effect of the filter on the image is shown in Fig. 7.

#### 2.1.2 Sliding reconstruction

A second approach to reconstruction, the sliding reconstruction method, was introduced by Murali^47^ and does not rely on Fourier analysis but on direct matching of the interference pattern.

The light intensity exiting the Savart plates can be written as

$$O(x,y)=S^{\prime }(x,y)=(\frac{1}{2})[S_{0}(x,y)F_{0}(x,y)+S_{1}(x,y)F_{1}(x,y)+S_{2}(x,y)F_{2}(x,y)+S_{3}(x,y)F_{3}(x,y)],$$ (3)

where O(x,y) is the intensity of the light, $S_{0}(x,y),S_{1}(x,y),S_{2}(x,y),S_{3}(x,y)$ are the Stokes components of the light entering the crystals, and $F_{0}(x,y),F_{1}(x,y),F_{2}(x,y),F_{3}(x,y)$ form the first row of the Mueller matrix elements.

**Fig. 8:**
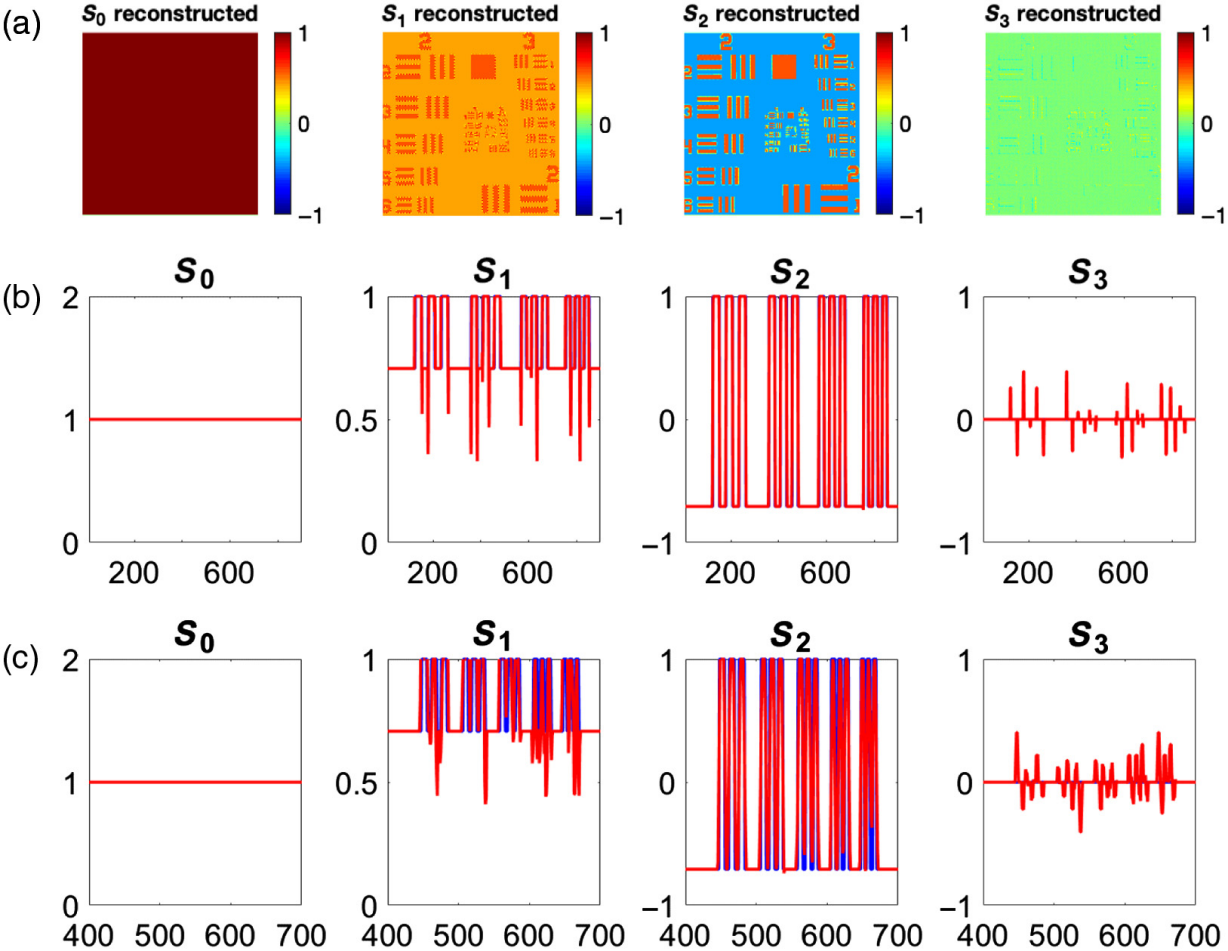
Stokes vector of a USAF target reconstructed with the sliding reconstruction method (a) showing traces of the (b) largest and (c) smallest blocks of resolution dashes. As can be observed from the changes from the raw signal to the reconstructed signal, strong discontinuities create artifacts in the Stokes images.

The Stokes components are estimated over the entire image. A unit cell, a 3×3 kernel in this case, is moved by one pixel either along the column or row. To compensate for multiple pixel calculations, the average value of the multiple reconstructions of the Stokes component is taken. Three reference images are needed for this reconstruction method: 0 ($S_{\mathrm{ref}0}$), 45 deg ($S_{\mathrm{ref}45}$), and right-hand circular ($S_{\text{refRHC}}$). The reconstruction using the sliding reconstruction method can be observed in Fig. 8.

The Stokes vectors produced using the reconstructions were used to populate the Mueller matrix in order to perform the MMD. This process has been extensively explained elsewhere.^35^^,^^42^^,^^48^

### 2.2 Anisotropic and *Ex Vivo* Biological Samples

The portable colposcope was first tested with optical elements of known Mueller matrices (air, linear polarizers) as well as an extruded silicone phantom and an *ex vivo* porcine cervix before being used in a pilot study in Mysore, India. The extruded silicone phantom consisted of a silicone strip with visible striations along the same direction. This sample has known polarimetric properties and is often used for device validation.^53^ The material’s transparency allows for minimal polarization information loss, due to its low absorption and scattering. The polarimetric system was also tested with an *ex vivo* paraffin-embedded porcine cervix—the embedding process can be found in detail elsewhere.^42^ The porcine cervix has a circumferentially aligned collagen structure around the os, similar to the human cervix, and therefore exhibits similar polarimetric properties.^42^ A Mueller matrix system utilized in a previous study was also used to validate our newly developed apparatus.^42^

### 2.3 Clinical Deployment

The evaluation of our system on healthy patients study was added to an ongoing screening protocol in a mobile clinic in Mysore, India. Patients were recruited among the ones coming for gynecological evaluation and Papanicolaou (Pap smear) testing. An IRB protocol (IRB-17-0181) was approved by Florida International University Institutional Review Board as well as the Public Health Research Institute of India’s Institution Ethics Review Board (2016-20-08-34) and informed consent was obtained from all subjects. A total of 22 study participants were recruited. Eligibility criteria included: (1) age≥18  years, (2) willing to undergo imaging, and (3) having the capacity to undergo informed consent process. Participants were screened for eligibility via chart review at the time of appointment. All eligible women were provided information about cervical cancer, study risks, and benefits. A brief data collection instrument was used to collect sociodemographic and medical information about each participant.

After recruitment, the patients underwent a standard gynecological exam that included a cervical inspection. For this purpose, a speculum was utilized to dilate the vaginal canal and access the cervix. Two strategies were devised for cervical visualization and positioning of the system for imaging. First, the system was mounted on a portable tripod that could be easily moved in front of the patient once the speculum was inserted. Second, the acquisition program was projected onto the screen of an iPhone 6 connected to the tripod. This was done with a commercially available app called Duet. This strategy is similar to having an eyepiece on a colposcope and allowed the system user to see and position the Savart system more efficiently rather than diverting his/her attention to a computer screen. The system was self-powered and required short acquisition time (∼1  s). The overall imaging portion of the study took about 10 min per patient. Little training was required in order to implement device deployment (less than 15 min). Finally, post-imaging, patients were administered the Pap smear.

## 3 Results

**Fig. 9:**
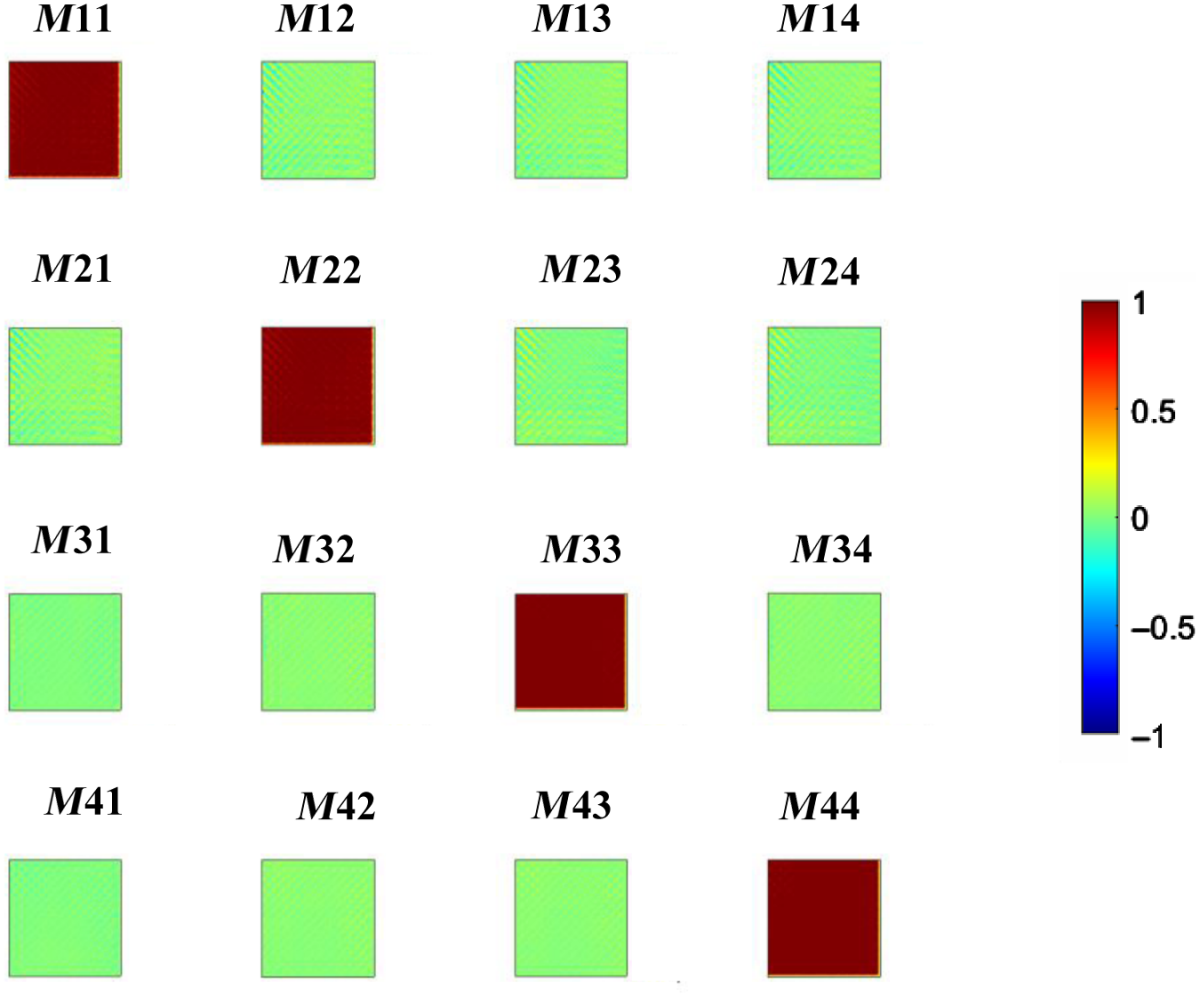
Mueller matrix of air. The portable device was tested in transmission to understand the error of the polarimetric response. Although some structural artifacts are present (small white lines in the matrix), the expected values of ones in main diagonal elements are present.

The error of the portable device was measured by taking the Mueller matrix of air. Figure 9 shows the Mueller matrix image of air obtained in transmission (light source positioned facing the PSA) displaying a typical unit matrix behavior. The total error in these images was well below 5%, but some structural error is perceivable in the images.

**Fig. 10:**
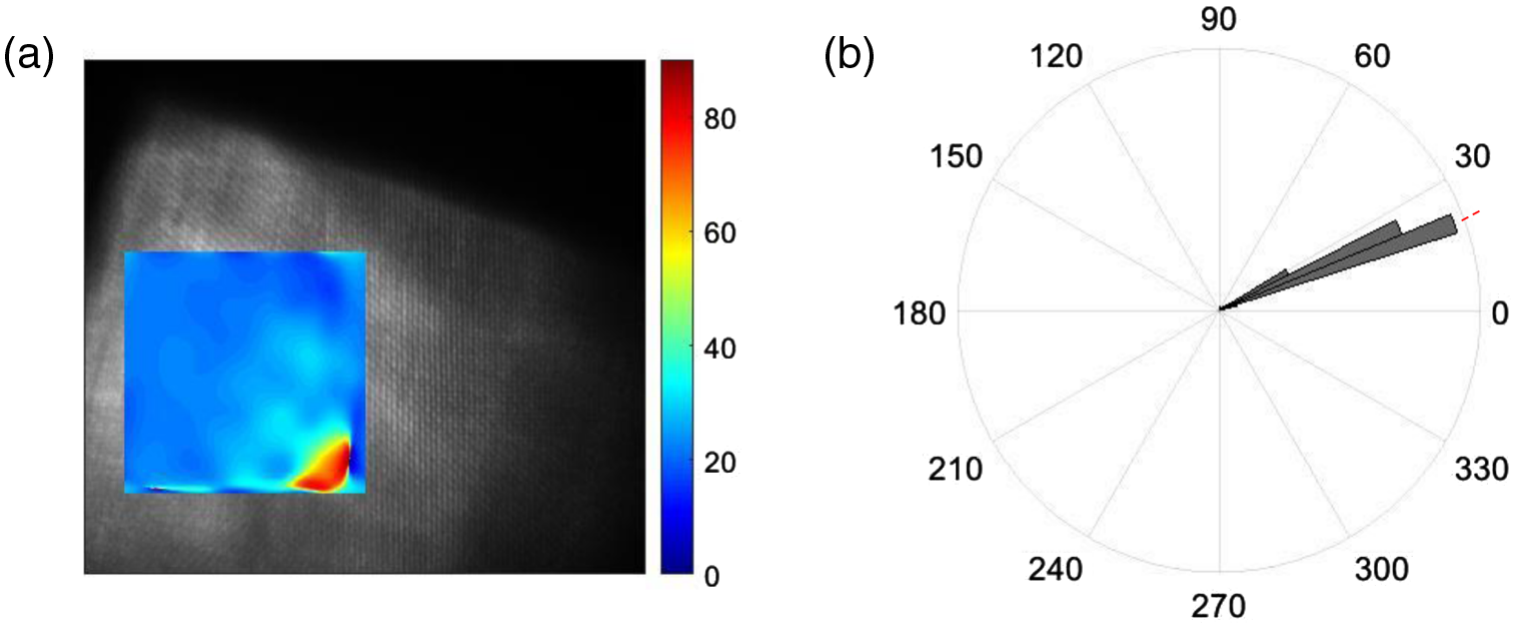
Extruded silicone sample recovered orientation. The silicone sample was placed at a 22-deg shift (marked in the red line) from the frame of reference. (a) Raw image (gray scale) with the recovered mean angle of a region of interest (colormap) and (b) circular histogram of the recovered angles.

The system was then tested with a silicone phantom. The phantom’s uniformity makes it a reliable target to validate the MMI system. The phantom was positioned at different orientations with respect to the system’s reference frame. Figure 10 displays the extruded silicone phantom shifted 22 deg from the reference frame. On the left [Fig. 10(a)], the raw image with the interferometric pattern can be seen in gray with the colored region of interest highlighted. The image on the right [Fig. 10(b)] shows the calculated angles (α), which were in agreement with the positioning of the phantom.

To understand the portable colposcope’s response to biological samples, the *ex vivo* paraffin-embedded porcine cervix was imaged. The zone of interest, which is the area between the os and the outer layer of the cervix, was determined by choosing a region of interest in the mid-section of the cervix. The os and the outer cervix have longitudinally aligned collagen and therefore are excluded from the polarimetric evaluation.

**Fig. 11:**
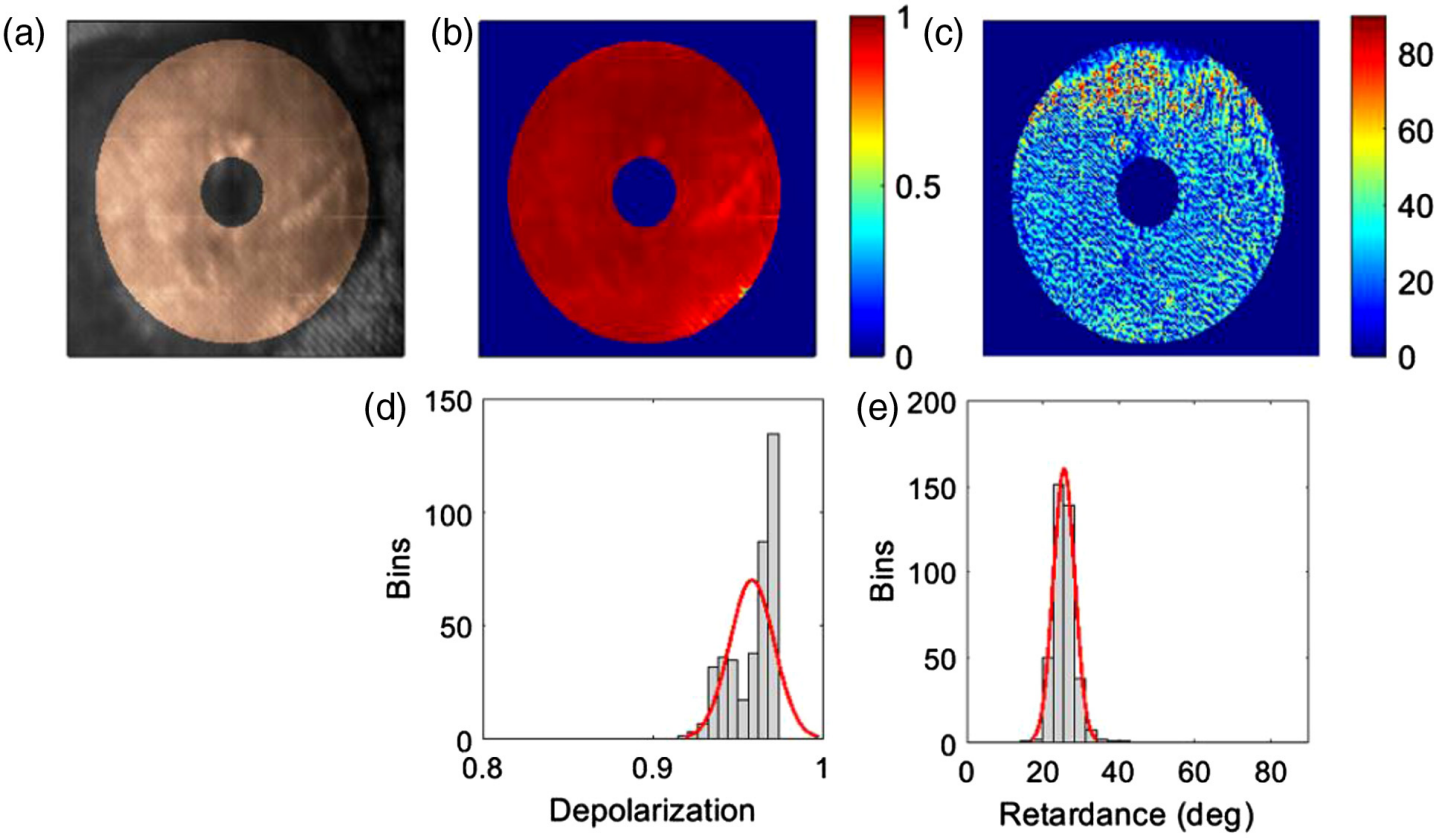
*Ex vivo* paraffin-embedded porcine cervix. (a) Raw image highlighting region of interest, (b) Mueller matrix decomposed depolarization, (c) Mueller matrix decomposed retardance, (d) distribution of depolarization, and (e) distribution of retardance angles.

Biological samples are strongly depolarizing due to their high scattering and absorbing nature. The polarized light interaction with collagen crosslinks also cause high retardance. Highlighting the zone of interest, we can observe a high level of depolarization (${\mathrm{MD}}_{\text{ave}}=0.96$) and retardance (${\mathrm{MR}}_{\text{ave}}=26\;\deg$) exhibited by the porcine cervix [Figs. 11(a)–11(c)], as we had expected. The depolarization is quantified from 0 to 1, going from low to high, respectively. The retardance is measured from 0 deg to 90 deg. Figures 11(d) and 11(e) portray the distribution of depolarization and retardance, respectively, which shows a similar outcome to what has been reported by Chue-Sang et al.^42^ for the paraffin-embedded porcine cervix.

**Fig. 12:**
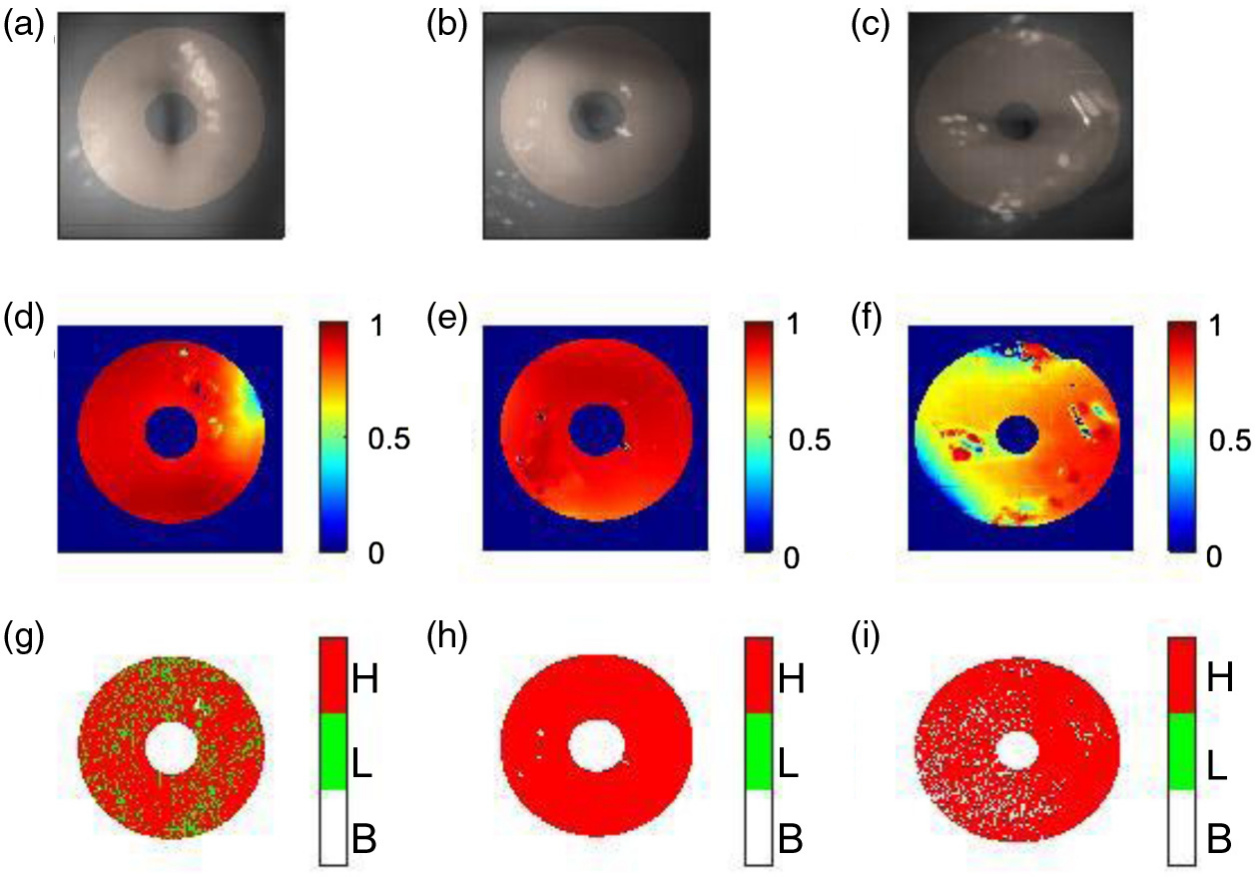
*In vivo* healthy human cervices with negative clinical evaluations (a)–(c) raw images with the highlighted region of interest, (d)–(f) the depolarization and (g)–(i) the retardance portrayed as a binary high (H, red)/low (L, green) with zero background (B, white) using a threshold of 25 deg.

The portable colposcope was further tested in a clinical pilot where *in vivo* human imaging took place. The average age of women imaged was 35±8  years old. Of the 22 patients imaged, six were deemed unsatisfactory due to image quality and therefore have been excluded from the resulting analysis. The remaining 16 patients received clinical assessments summarized in Table 1. Three patient images and corresponding MMDs are reported in Fig. 12. The zone of interest is highlighted superimposed over the raw images (a)–(c). The three cervices show a high level of depolarization (d)–(f) and retardance (g)–(i), as expected of healthy tissue.

**Table 1:** Patient age and clinical evaluation performed by a physician.

Patient #	Age	Clinical evaluation/Pap smear
1	50	Polyp (green)
2	30	Negative (gray)
3	45	Negative (gray)
4	36	Negative (gray)
5	33	Negative (gray)
6	37	Neutrophils (cyan)
7	30	Negative (gray)
8	23	Negative (gray)
9	29	Inflammatory (red)
10	29	Inflammatory (red)
11	34	Negative (gray)
12	50	Atrophic with inflammation (purple)
13	40	Inflammatory (red)
14	40	Inflammatory (red)
15	28	Negative (gray)
16	30	Negative (gray)

The region of interest along the circumferential zone can be seen superimposed in the raw images (Fig. 12). The devices exhibit high depolarization and retardance values, as have been reported for healthy cervix tissue.

**Fig. 13:**
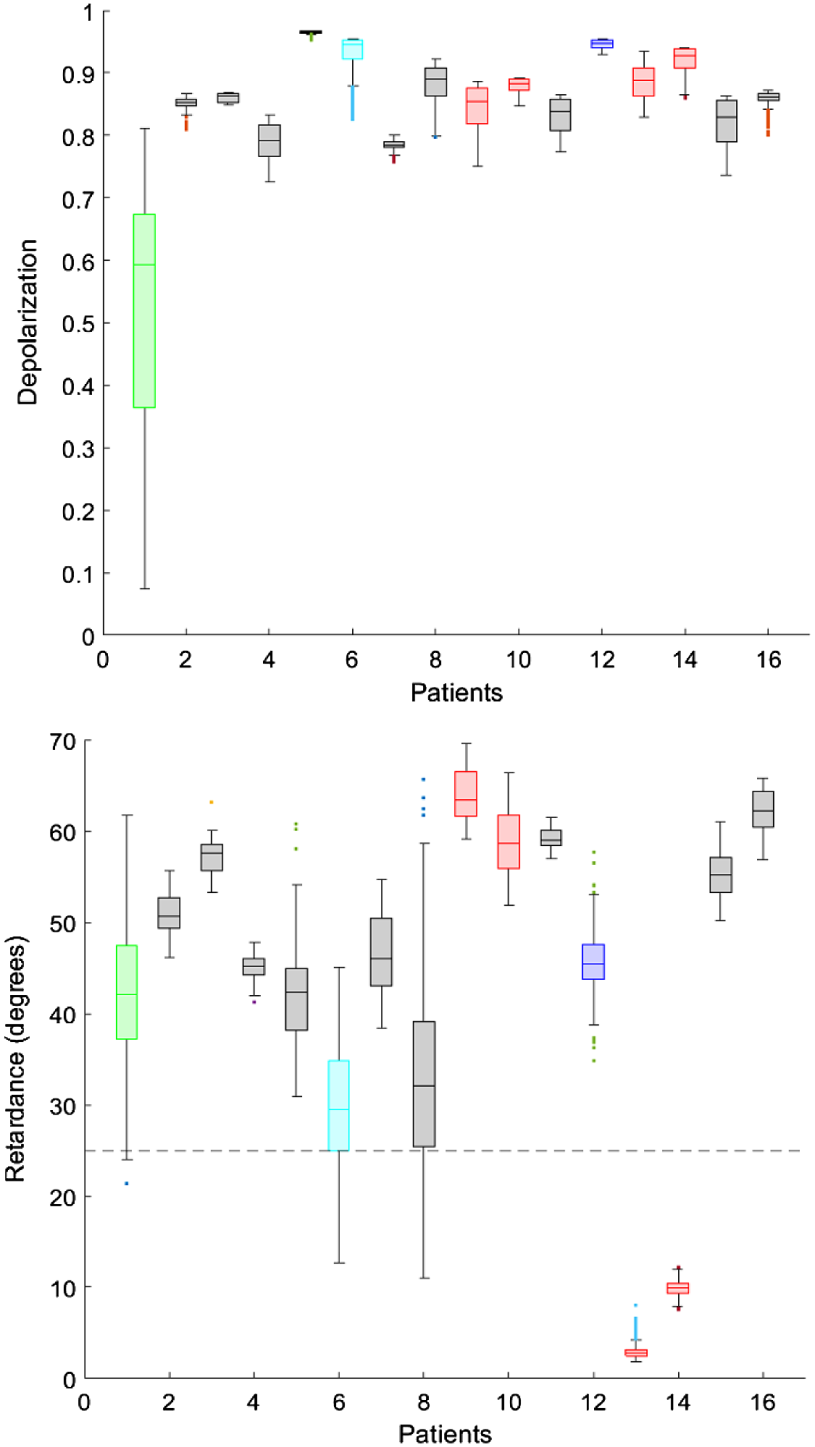
The depolarization (top) and retardance (bottom) of the 16 healthy subjects are summarized. The healthy cervices show a pattern of high depolarization, with the lowest value (patient 1) being the polyp. The retardance shows a trend of high values as well, with only two cervices having a median value below the 25 degree threshold.

A summary of all the 16 patients imaged is displayed in Fig. 13. A region of interest has been selected to derive the depolarization and retardance information. The data have been color-coded in reference with Table 1 to aid in understanding of the polarimetric behavior compared to the clinical evaluation.

The 16 cervices showed a trend of high (median>0.78) depolarization with the exception of patient 1, which had a polyp. The collagen structure in polyps differs from normal stroma, which was perceived by a lower depolarization value, as has been previously described by others.^54^ The retardance (measured in deg) is visualized as high (red) and low (green), with a threshold of 25 deg—approach was first introduced by Rehbinder et al.^39^ The retardance values had an overall high trend (median >29  deg), with the exception of patient 13 and 14.

## 4 Discussion

We have introduced a new snapshot Mueller matrix polarimeter capable of the fast acquisition of a full Mueller matrix. The imaging was performed in healthy cervices, where there were no cases of dysplasia. The results of the MMD support this clinical evaluation by showing a high depolarization trend with an average depolarization value of 0.85, where the lowest value, 0.52, is displayed by region of interest with a polyp. This is in accordance with previously reported work, where a polyp changes the collagenous structure and therefore the polarimetric response.^37^ The retardance shows an average of 44 deg, which is within the range found in literature for healthy cervices. Some non-uniformity of the retardance data can be due to the heterogeneity of the cervix as well as presence of artifacts such as specular reflection.

The ability of MMI to identify differences in the collagen’s polarimetric response by detecting the distinct depolarization response between the polyp and other healthy cervices shows how our portable system has the potential to be deployed for use in conjunction with routine cervical screening. The system is fully powered by a laptop computer and can be deployed in conditions where electrical outlets are not readily available. The cost of the system is also relatively low compared to current colposcopes (∼$2000), with a limiting factor being the cost of the Savart plates (∼$200). It is to be noted, however, that should the modality prove to be useful in sensing dysplastic lesions, higher production levels could be considered, lowering the overall production cost.

Further studies are necessary to truly determine the diagnostic power of this approach. One noted issue with the current system is the size of the illuminator and the ability of all illumination sources to reach the cervix without cutoff from the uterine walls. Six sets of patient images, out of the 22 patients imaged, were discarded due to absence of data on account of poor illumination from one or more polarization states. In older patients and women that have experienced multiple pregnancies, this effect could be more severe limiting the use of our apparatus. To minimize this effect, we are currently redesigning the illuminator to allow for more direct access to the cervix. A second issue noted is the development of artifacts in the Fourier-based analysis due to strong discontinuities in the inverse Fourier transform. When the regions of interest laid around those problematic regions, the second analysis (sliding reconstruction method) was utilized. Finally, another limiting factor was the loss of fringe contrast suffered in regions with high curvature. This, at times, limited the area that could be analyzed, and future work will focus on optimizing the imaging optics so that the entire cervix can be processed. Future studies will also be targeted toward testing the device’s ability to detect malignancies in cervical tissues.

## 5 Conclusion

A feasibility study was conducted among healthy volunteers in Mysore, India. The results showed accordance with current literature about the depolarization and retardance behavior of healthy cervices.^32^^,^^37^^,^^39^^,^^44^ Due to the dependence spatial interference pattern of the current methodology, there are limitations on the deployment and data analysis, as well as the limitation of non-real-time feedback. Recent advancement in polarization technology may lead to new direction of this research. Particularly, new cameras with integrated polarization capability (4-Directional Wire Grid Polarizer Array such as the Sony’s IMX250MZR CMOS chip) may allow for fast acquisition of four linear states (horizontal, vertical, 45 deg polarization, and −45  deg polarization). This technology would allow at maximum the creation of a reduced Mueller matrix (i.e., not 4×4), requiring other data analysis techniques. In that regard, the proposed Savart method is still seen as superior as it offers the capability of capturing a full Mueller matrix.

In conclusion, we have developed a portable MMI system that can be deployed in the low-resource setting for cervical imaging. We believe that this type of imagery could improve the cervical screening assessment in the low-resource setting beyond the current procedures.
